# Relationship between Decreased Serum Superoxide Dismutase Activity and Metabolic Syndrome: Synergistic Mediating Role of Insulin Resistance and *β*-Cell Dysfunction

**DOI:** 10.1155/2020/5384909

**Published:** 2020-06-12

**Authors:** Yiwen Liu, Chifa Ma, Lu Lv, Pingping Li, Chunxiao Ma, Shuli He, Jingbo Zeng, Fan Ping, Huabing Zhang, Wei Li, Lingling Xu, Yuxiu Li

**Affiliations:** ^1^Department of Endocrinology, Key Laboratory of Endocrinology, Ministry of Health, Peking Union Medical College Hospital, Peking Union Medical College, Chinese Academy of Medical Sciences, Beijing 100730, China; ^2^State Key Laboratory of Bioactive Substance and Function of Natural Medicines, Institute of Materia Medical Sciences and Peking Union Medical College, Beijing 100050, China; ^3^Diabetes Research Center of Chinese Academy of Medical Sciences, Beijing 100050, China; ^4^Department of Nutrition, Peking Union Medical College Hospital, Beijing 100730, China; ^5^Department of Endocrinology, Fuxing Hospital, The Eighth Clinical Medical College, Capital Medical University, Beijing 100038, China

## Abstract

The interplays of cellular aging and oxidative stress (OS) markers form a complex network, which has been reported to be interrelated with numerous age-related and metabolic diseases, including metabolic syndrome (MS). However, given the multifactorial mechanisms of MS, several important confounders such as dietary factors and the reciprocal effect among these markers have not been considered and adjusted in previous investigations regarding the associations of cellular aging and OS markers with MS and its related metabolic abnormalities. To explicate this, we conducted a cross-sectional study among 533 Chinese adults. All the participants underwent a 75 g oral glucose tolerance test. Dietary data were collected via a 24-hour dietary recall and subsequently analyzed by a registered dietitian using nutrition calculation software. Clinical diagnosis of MS was made according to the revised National Cholesterol Education Program Adult Treatment Panel III criteria (2004) with waist circumference cutoff modified for an Asian population. The leukocyte telomere length, mitochondrial DNA copy number, 8-hydroxy-2-deoxyguanosine, superoxide dismutase (SOD) activity, and glutathione reductase were examined. SOD activity was significantly decreased in MS subjects (62.06 ± 16.89 U/mL vs. 56.25 ± 22.61 U/mL, *P* = 0.001) and exhibited a descending trend across sequential increase of MS component number (*P* for trend = 0.031). SOD activity is modestly correlated with glucose indicators and insulin sensitivity and *β*-cell function indices and was independently and negatively correlated with the level of triglyceride. An independent association between SOD activity and MS was observed after adjusting for metabolic indicators, dietary factors, cellular aging, and OS markers, as well as insulin sensitivity and *β*-cell function indices. However, the statistical significance of the association between SOD activity and MS was attenuated after adjusting for the Matsuda insulin sensitivity index (ISIM) and insulin secretion-sensitivity index-2 (ISSI-2), suggesting a possible mediating effect. Therefore, we conducted a mediation model analysis, which showed that decreased ISIM and ISSI-2 partially and synergistically mediated the contribution of decreased SOD activity to MS. In conclusion, decreased SOD activity is an independent predictor for increased risk of MS, and insulin resistance and *β*-cell dysfunction partially mediate the relationship between decreased SOD activity and MS.

## 1. Introduction

Metabolic syndrome (MS) is characterized as a cluster of multiple metabolic abnormalities including central obesity, hypertension, dyslipidemia, and hyperglycemia, which contributes to aging and numerous age-related diseases including type 2 diabetes (T2D), cardiovascular diseases, and premature mortality [1]. The underlying mechanisms are multifactorial, among which insulin resistance plays a pivotal role [2]. In recent years, oxidative stress (OS) has been intensively investigated and provided important mechanistic insights into the pathogenesis of MS and its related metabolic abnormalities [2–6]. As important contributors of aging and age-related diseases, the associations between cellular aging markers (e.g., leukocyte telomere length (LTL) [7] and mitochondrial DNA copy number (mtDNAcn) [8]) and MS have also been increasingly explored in prior population-based studies, which however have yielded contradictory results in terms of their causal relationships [9–13]. The interplay between cellular aging and OS constitutes a complex regulatory network [14], participating in pathogenesis of a multitude of age-related diseases. Nevertheless, their interactions and mutual confounding effects were scarcely taken into consideration and adjusted in previous investigations regarding the associations between these markers and MS. Additionally, given that dietary factors (e.g., calorie intake, fat intake, and dietary antioxidants) may have a profound effect on cellular aging and OS markers as well as MS [15–17], it is necessary to eliminate the confounding effects of dietary factors when investigating the associations of cellular aging and OS markers with MS, whereas data of diet were not obtained in previous studies. Additionally, the interrelations among cellular aging and OS markers, insulin resistance, and *β*-cell dysfunction in MS subjects were sparsely examined in previous studies. Therefore, in the present study, we sought to investigate the cross-sectional relationships of cellular aging and OS markers with MS and its related metabolic abnormalities after adjusting for the potential confounding effect of the interrelations of these markers and dietary factors and explicate the potential role of insulin resistance and *β*-cell dysfunction in the interactions of cellular aging and OS markers with MS and its related metabolic abnormalities.

## 2. Methods and Materials

### 2.1. Study Participants

A cross-sectional population comprising 582 adults was obtained from Changping suburb of Beijing, China, between March 2014 and January 2015. The study protocols had been approved by the Ethics Committee of Peking Union Medical College Hospital. All participants have provided written informed consent forms. To eliminate the interference of lipid-lowering drugs and hypoglycemic agents on the MS categorization of the study participants and evaluation of insulin sensitivity and *β*-cell function, data from 49 individuals taking lipid-lowering drugs or/and hypoglycemic agents were excluded. Therefore, a total of 533 adults were included in the subsequent data analysis of the current study.

### 2.2. Physical Examinations

The anthropometric data including height, weight, waist circumstance (WC), hip circumstance (HC), systolic blood pressure (SBP), and diastolic blood pressure (DBP) were collected by trained clinicians. Body mass index (BMI) was calculated as body weight (in kilograms) divided by the square of height (in meters). WC was measured exactly at midway between the peak of the iliac crest and lowest rib. HC was measured exactly at the level of trochanters. BP was measured with a standard method.

### 2.3. Diagnostic Criteria

The clinical diagnosis of MS was made according to the revised National Cholesterol Education Program Adult Treatment Panel III criteria (2004) [18] with waist circumference cutoff modified for an Asian population [19]. The presence of any 3 of the following 5 criteria constitutes MS diagnosis: (1) elevated WC: ≥90 cm in males and ≥80 cm in females; (2) elevated TG: ≥1.7 mmol/L; (3) reduced HDL-C: <1.0 mmol/L in males and <1.3 mmol/L in females; (4) elevated BP: SBP ≥ 130 mmHg and/or DBP ≥ 85 mmHg or under antihypertensive drug treatment in a patient with a history of hypertension; and (5) elevated fasting glucose: fasting plasma glucose (FPG) ≥ 5.6 mmol/L.

### 2.4. Dietary Data Collection

The details of dietary data collection have been described elsewhere [20]. Briefly, data collected via a 24-hour dietary recall were analyzed by a registered dietitian using nutrition calculation software developed by registered dietitians based on the Microsoft Office Access 2007 database. Subsequently, the calculations of dietary component intake were performed based on the China Food Composition (2004), including calories, fat, carbohydrate, protein, vitamin A, carotene, vitamin C, vitamin E, zinc, selenium, and manganese.

### 2.5. Biochemical Analysis

Venous blood was collected in the morning after an overnight fast for at least 10 hours. Study participants underwent a 2-hour 75 g oral glucose tolerance test (OGTT), and plasma glucose (PG) and serum insulin (INS) were measured at four time points including 0 minutes (min), 30 min, 60 min, and 120 min. PG was determined using the glucose oxidase assay. The chemiluminescence immunoassay was applied for the detection of INS (#02230141 (128434), Siemens Medical Solutions Diagnostics, Tarrytown, NY, USA). The fasting venous blood samples were used to determine levels of glycosylated hemoglobin (HbA1c) by high-performance liquid chromatography, as well as uric acid (UA), total cholesterol (TC), total triglyceride (TG), high-density lipoprotein cholesterol (HDL-C), and low-density lipoprotein cholesterol (LDL-C) using an automatic analyzer.

### 2.6. Assessment of Insulin Sensitivity and *β*-Cell Function

Two fasting indices (homeostatic model assessment of insulin resistance (HOMA-IR) and *β*-cell function (HOMA-*β*)) and two OGTT-derived indices (Matsuda insulin sensitivity index (ISIM) and insulin secretion-sensitivity index-2 (ISSI-2)) were calculated as originally described [21–23]. HOMA-IR and ISIM were used to evaluate the insulin sensitivity whereas HOMA-*β* and ISSI-2 were used to evaluate *β*-cell function.

### 2.7. Measurements of Cellular Aging and OS Markers

Cellular aging markers including LTL and mtDNAcn were determined using quantitative polymerase chain reaction (qPCR) which has been described in detail in our previous publications [24, 25]. LTL was calculated as the relative ratio of telomere repeat copy number to the single copy number according to the monochrome multiplex qPCR protocol. And the relative mtDNAcn value was adjusted by simultaneous measurement of nuclear DNA. The measurements of OS markers including 8-hydroxy-2-deoxyguanosine (8-OHdG), superoxide dismutase (SOD) activity, and glutathione reductase (GR) activity were performed using enzyme-linked immunosorbent assay kits according to the manufacturer's instructions (Cloud-Clone Corp, Houston, USA).

### 2.8. Statistical Analysis

All the analyses were performed using SPSS.26.0 (IBM) and GraphPad Prism 8.1.1 (GraphPad Software, Inc.). Normally distributed continuous variables are presented as a mean ± standard deviation (SD), and nonnormally distributed continuous variables are presented as a median (interquartile range), whereas categorical variables are presented as a number (percentages). Normality transformations were performed on the skewed distributed variables when necessary. For continuous variables, Student's *t*-test or nonparametric Mann-Whitney *U* test was conducted for comparison of two groups, where appropriate. The linear trend of variables was tested using one-way analysis of variance (ANOVA) for normally distributed variables or Jonckheere's trend test for nonnormally distributed variables. Pearson correlation analysis was performed to explore the unadjusted bivariate correlations, and subsequently, partial correlation analysis was implemented to examine the bivariate correlations adjusting for potential confounders. Stepwise multivariate logistic regression model analysis was implemented to identify potential independent predictors for MS. Prior to the inclusion of covariates into the multivariate logistic regression model, their multicollinearity was examined, and thus, the variables with a variance inflation factor > 5 were excluded. To identify potential mediators of the association between the independent variable and the dependent variable, mediation model analyses were conducted using Hayes' Model 6 in the PROCESS macro Version 3.3 for SPSS. The total effect, which referred to the original relationship between the independent variable and the dependent variable, was divided into an indirect effect and a direct effect. The indirect effect, also called mediation effect, could be interpreted as the association between the independent variable and the dependent variable that could be explained by the difference in mediators, whereas the direct effect referred to the effect that remained significant after controlling for the mediators. The absence of “0” in the 95% confidence interval indicated a statistical significance in the mediation analyses. In all analyses, a *P* value < 0.05 (2-tailed) was considered as statistically significant.

## 3. Results

### 3.1. Differences of Metabolic Profiles and Dietary Intake between Individuals with and without MS

Table 1 reveals the differences of baseline metabolic profiles and dietary intake between individuals with and without MS. A total of 533 adults (34.5% male) aged 19-90 years were included in the subsequent analysis and categorized into non-MS (*n* = 247) and MS (*n* = 286). Not surprisingly, the individuals with MS exhibited a female predominance; an older age as well as a poorer metabolic profile including significantly higher BMI, WC, HC, BP, HbA1c, PG at all time points in OGTT, and UA; and a worsened lipid profile characterized by elevated TG and LDL-C and reduced HDL-C. The significantly higher HOMA-IR as well as lower ISIM and ISS-2 in MS subjects indicated a lower level of insulin sensitivity and *β*-cell function. In terms of the differences of dietary intake, the total intake of calories did not differ between individuals with and without MS whereas the calorie intake per kilogram body weight was lower in MS subjects than those in non-MS subjects. And the differences of other dietary components did not reach a statistical significance.

### 3.2. SOD Activity Decreased in MS Subjects and Showed a Linear Descending Trend across the Increase of MS Component Number

**Figure 1:**
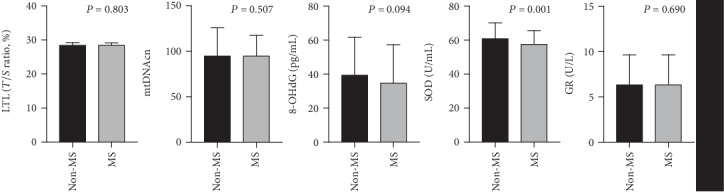
Differences of cellular aging and oxidative stress markers between individuals with and without metabolic syndrome. SOD activity was significantly decreased in MS subjects compared with non-MS subjects (*P* = 0.001), whereas differences of other indicators did not reach the statistical significance. The *P* value for LTL and mtDNAcn was age-adjusted. Abbreviations: LTL: leukocyte telomere length; mtDNAcn: mitochondrial DNA copy number; 8-OHdG: 8-hydroxy-2-deoxyguanosine; SOD: superoxide dismutase; GR: glutathione reductase.

**Figure 2:**
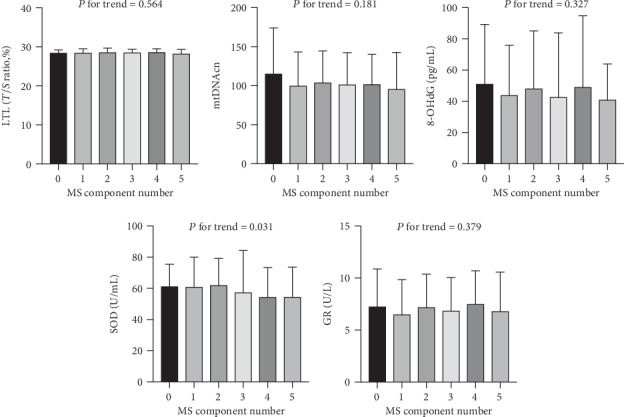
Linear trend tests for cellular aging and oxidative stress markers across metabolic syndrome component numbers. mtDNAcn and SOD activity exhibit a linear descending trend along with the progressive increase of MS component number, whereas other indicators did not show a statistically significant trend. Abbreviations: LTL: leukocyte telomere length; mtDNAcn: mitochondrial DNA copy number; 8-OHdG: 8-hydroxy-2-deoxyguanosine; SOD: superoxide dismutase; GR: glutathione reductase.

As indicated in Figure 1, the SOD activity of MS subjects was significantly reduced compared with that of non-MS subjects (*P* = 0.001), whereas age-adjusted LTL, age-adjusted mtDNAcn, 8-OHdG, and GR were comparable between individuals with and without MS. As shown in Figure 2, the SOD activity (*P* for trend = 0.031) showed a linear downward trend across the progressive increase of the MS component number, whereas the linear trend of change of LTL, mtDNAcn, 8-OHdG, and GR did not reach statistical significance.

### 3.3. Independent and Negative Correlation between SOD Activity and Level of TG

**Figure 3:**
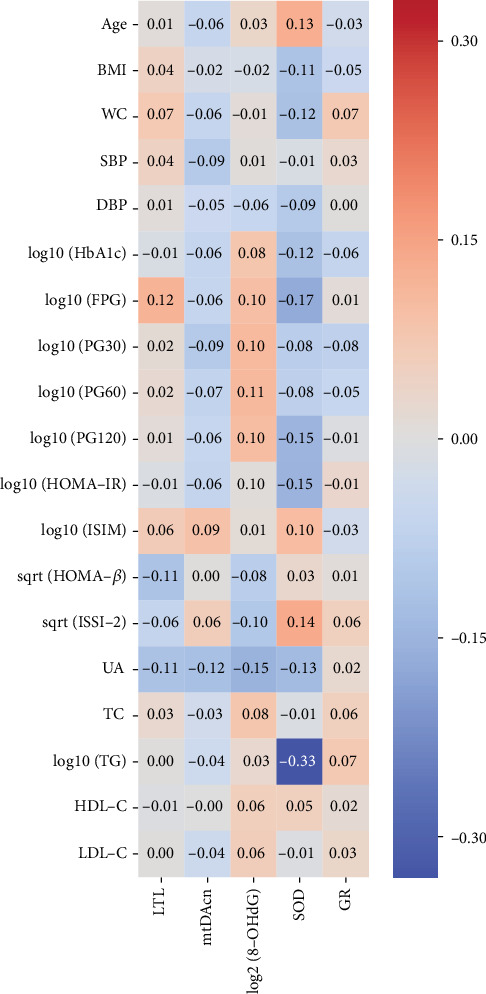
Pearson correlation heat map between cellular aging, oxidative stress, and inflammatory markers and clinical characteristics. The figures on the heat map represent the Pearson *r*. ∣*r* | >0.08, *P* < 0.05; ∣*r* | >0.11, *P* < 0.01. The values of the color bar represent the correlation coefficients (Pearson *r*). The red color means a positive correlation whereas the blue color means a negative correlation. And the depth of the color represents the size of the correlation coefficient. Darker color represents a stronger correlation whereas lighter color represents a weaker correlation. Abbreviations: BMI: body mass index; WC: waist circumference; SBP: systolic blood pressure; DBP: diastolic blood pressure; HbA1c: glycosylated hemoglobin A1c; FPG: fasting plasma glucose; PG30, 60, and 120: postload plasma glucose at 30 min, 60 min, and 120 min during OGTT; ISIM: Matsuda index; ISSI-2: insulin secretion-sensitivity index-2; UA: uric acid; TC: total cholesterol; TG: total triglyceride; HDL-C: high-density lipoprotein cholesterol; LDL-C: low-density lipoprotein cholesterol; LTL: leukocyte telomere length; mtDNAcn: mitochondrial DNA copy number; 8-OHdG: 8-hydroxy-2-deoxyguanosine; SOD: superoxide dismutase; GR: glutathione reductase.

Pearson correlations of cellular aging and OS markers with metabolic profiles are presented in Figure 3. A moderate, negative correlation between the SOD activity and TG was observed (*r* = −0.33, *P* < 0.01). Additionally, SOD activity was also inversely correlated with HbA1c, FPG, PG120, UA, and HOMA-IR (*r* = −0.12, -0.17, -0.15, -0.13, and -0.15, *P* < 0.01, respectively) and positively correlated with ISIM and ISSI-2 (*r* = 0.10, *P* < 0.05, and *r* = 0.14, *P* < 0.01, respectively), which however were somewhat weak. In the subsequent partial correlation analysis, after adjusting for potential confounders including gender, age, BMI, WC, SBP, DBP, HbA1c, PG at all time points during OGTT, UA, HDL-C, LDL-C, dietary factors (dietary intake of calories per kilogram body weight, fat, vitamin A, carotene, vitamin C, vitamin E, zinc, selenium, and manganese), and cellular aging and OS markers, the correlation between SOD activity and TG was not attenuated (*r* = −0.30, *P* < 0.001), indicating an independent association between SOD activity and TG.

### 3.4. SOD Activity Was an Independent Predictor for Risk of MS

To examine whether SOD activity could independently predict MS, a stepwise multivariate logistic regression analysis was performed. As shown in Table 2, the higher level of SOD activity remained independently associated with a lower risk of MS after progressively adjusting for gender, age, and BMI (OR for 1SD increase of SOD = 0.719, 95% CI 0.572-0.903, *P* = 0.005, model 1), dietary intake of calories per kilogram body weight and fat as well as dietary antioxidants (OR for 1SD increase of SOD = 0.721, 95% CI 0.569-0.914, *P* = 0.007, model 2), and cellular aging and OS markers (OR for 1SD increase of SOD = 0.718, 95% CI 0.565-0.912, *P* = 0.007, model 3), suggesting the independent predictive role of SOD activity for MS. In model 4, ISIM and ISSI-2 were further adjusted based on model 3, and statistical significance of the association between SOD activity and MS was attenuated (OR for 1SD increase of SOD = 0.750, 95% CI 0.576-0.978, *P* = 0.034), suggesting a possible mediating effect of insulin sensitivity and *β*-cell function on the link between SOD activity and MS.

### 3.5. Insulin Sensitivity and *β*-Cell Function Partially and Synergistically Mediated the Relationship between SOD Activity and MS

**Figure 4:**
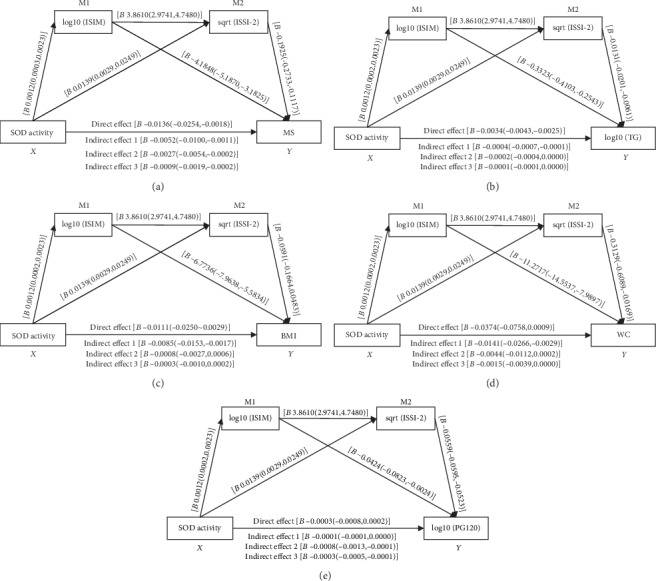
Mediation model analysis of the association among SOD activity, ISIM, ISSI-2, and MS as well as MS-related metabolic abnormalities. *X* represents independent variable (SOD activity); *Y* represents dependent variable ((a) MS; (b): log(TG); (c) BMI; (d) WC; (e) log(PG120)); M1 and M2 represent mediating variables 1 (log10(ISIM)) and 2 (sqrt(ISSI-2)); indirect effects 1, 2, and 3 represent the indirect effect of SOD → log10(ISIM) → *Y*, SOD → sqrt(ISSI‐2) → *Y*, and SOD → log10(ISIM) → sqrt(ISSI‐2) → *Y*, respectively. The effect was presented as *B* (95% CI). The absence of “0” in the 95% CI represents the statistical significance of the effect. The indirect effects 1, 2, and 3 and the direct effect are significant in (a). The indirect effect 1 is significant in (b–d) whereas the indirect effects 2 and 3 are significant in (e). Abbreviations: *B*: *β*-coefficient; 95% CI: 95% confidence intervals; SOD: superoxide dismutase; ISIM: Matsuda insulin sensitivity index; ISSI-2: insulin secretion-sensitivity index-2; MS: metabolic syndrome; TG: triglyceride; BMI: body mass index; WC: waist circumference; PG120: postload plasma glucose at 120 min during OGTT.

Long-term insulin resistance could trigger *β*-cell dysfunction [26, 27]. Both insulin resistance and *β*-cell dysfunction have been demonstrated to be established pivotal mechanisms of MS [2, 26], and an impaired antioxidant defense system has been reported to contribute to the development of MS [4]; therefore, based on the finding of the multivariable logistic regression analysis in this study, we hypothesized that impaired insulin and *β*-cell dysfunction might mediate the effect of the decrease of SOD activity on the development of MS and its related metabolic abnormalities. Thus, mediation model analyses were performed, and the results are displayed in Figure 4. As expected, Figure 4(a) suggests that impaired insulin sensitivity and *β*-cell dysfunction showed a partial and synergistic mediating effect on the relationship between the decrease of SOD activity and MS. Subsequently, as indicated in Figures 4(b)–4(d), the relationship between the decrease of SOD activity and the elevation of TG, BMI, and WC was mediated by impaired insulin sensitivity rather than *β*-cell dysfunction. In contrast, as shown in Figure 4(e), the relationship between the decrease of SOD activity and the elevation of PG120 was mediated by *β*-cell dysfunction rather than insulin resistance. These findings suggested the different dominant mechanisms for different MS-related metabolic abnormalities.

## 4. Discussions

To the best of our knowledge, this is the first study to explore the association between SOD activity and MS and its related metabolic abnormalities adjusting the confounding effect of cellular aging and OS markers, as well as the dietary factors. Our findings suggested that decreased SOD activity was associated with increased MS independently of cellular aging and OS markers, as well as the dietary risk factors, which was partially and synergistically mediated by impaired insulin sensitivity and *β*-cell dysfunction.

Several previous cross-sectional studies have compared the antioxidant defense system of subjects with and without MS, however, yielded mixed results [6, 28–32]. We observed a decrease of SOD activity in MS subjects, supporting the notion that antioxidant defense capability was impaired in MS subjects [4], although the alteration of the absolute level of SOD activity seems weak, which could limit the discriminatory performance of the SOD activity in differentiating MS from non-MS. However, we failed to find alteration of another fundamental antioxidant enzyme GR activity in MS subjects. More antioxidants need to be measured to comprehensively evaluate the antioxidant defense capacity. Our finding of a linear descending trend of SOD activity across the sequential increase of the MS component number is in line with the observation of a previous investigation [33]. LTL and mtDNAcn have also been reported to exhibit such a linear descending trend previously [13, 34]; however, we failed to observe a linear trend of these cellular aging markers. In the present study, we observed a significant negative correlation between SOD activity and TG as well as blood glucose indicators (HbA1c, FPG, and 2hPG); however, the absence of correlations of cellular aging markers and OS markers with other MS-related metabolic indicators such as BMI, WC, BP, and HDL-C disaccords with the previous findings [28, 33, 35]. A possible explanation for these discrepancies might be attributed to the different populations of the different studies. The cross-sectional association between SOD activity and MS has been demonstrated in several prior publications [31, 33]. However, our study is the first to explicate the independent association between SOD activity adjusting for the numerous metabolic, dietary, and cellular aging and OS confounders. Notably, this is the first study to explore the correlations between cellular aging as well as OS markers and the OGTT-derived indices of insulin sensitivity and pancreatic *β*-cell function including ISIM and ISSI-2. And we observed a modest correlation between SOD activity and ISIM as well as ISSI-2, indicating the association between decreased SOD activity and insulin resistance as well as *β*-cell dysfunction. Compared with HOMA-IR, which represents peripheral or hepatic insulin sensitivities based on the assumption that they are equivalent, ISIM has been demonstrated to reflect whole-body insulin sensitivity for its strong correlation with the gold standard whole-body insulin sensitivity index derived from euglycemic insulin clamp [22, 36]. And HOMA-*β* is a rough indicator of baseline or fasting insulin secretion, whereas ISSI-2 is a validated OGTT-based analogue to the actual disposition index from the intravenous glucose tolerance test [23], which reflects the *β*-cell compensatory capacity. In the development of MS, the decrease of glucose-stimulated insulin secretion usually appears earlier and is more severe, whereas the baseline insulin secretion could not be impaired or even increase before the development of type 2 diabetes [37]. Therefore, our observation of a decreased ISSI-2 and unaltered HOMA-*β* reflects the impairment of glucose-stimulated insulin secretion and reservation of baseline insulin secretion, which is consistent with the metabolic syndrome.

It is noteworthy that the association between the decrease of SOD activity and MS was found independent of dietary intake of calories, fat, dietary antioxidants, as well as cellular aging and OS markers. This finding indicates that SOD activity could act as an independent predictor for the prevalence of MS. As a canonical antioxidant enzyme, SOD is able to catalyze the conversion of O_2_^·^ to H_2_O_2_, scavenging the reactive oxygen species to prevent OS [4]. Thus, the decrease of SOD activity could trigger OS. The interconnections among OS and cellular aging form a complex regulation network [14], which has been demonstrated as a trigger as well as an outcome of MS and its related metabolic abnormalities [4]. Our current finding might suggest that the association between SOD activity and MS could not be explained by pathways involved with cellular aging.

Despite that the exact underlying mechanism is not yet fully understood, MS has been demonstrated as a multifactorial disease with numerous mechanisms, among which insulin resistance and *β*-cell dysfunction play an important role [2, 26]. OS has been implicated in the development of insulin resistance [38] and *β*-cell dysfunction [39]. In the current study, the mediation model showed that impaired insulin sensitivity and *β*-cell dysfunction partially and synergistically mediate the association between decreased SOD activity and MS, suggesting that the link between decreased SOD activity and MS could be partially explained by the impaired insulin sensitivity and *β*-cell dysfunction. In terms of individual MS-related metabolic abnormalities, the relationship between decreased SOD activity and elevated TG, obesity, and central obesity was merely mediated by insulin resistance, whereas the contribution of decreased SOD activity to elevated postload blood glucose was merely mediated by *β*-cell dysfunction. These findings support the notion that the Chinese population tends to exhibit higher insulin sensitivity but more vulnerable *β*-cell function in the development of type 2 diabetes [40], whereas hypertriglyceridemia as well as central obesity are indicators of insulin resistance [41].

Overall, several strengths deserved to be addressed. First, when explicating the relationship between cellular aging markers and OS markers and MS as well as its related metabolic abnormalities, we adjusted the potential confounding effect of dietary risk factors for MS and the mutual effect of these markers. Second, all study participants have undergone 2 h OGTT, and therefore, we are the first to explore the association of cellular aging markers and OS markers with OGTT-stimulated indices of insulin sensitivity and *β*-cell function. Nevertheless, there are some limitations as well. The cross-sectional design limited our ability to explicit the causal relationship between SOD activity and MS and its related metabolic abnormalities. Furthermore, all the study participants were recruited from a single area of a city, suggesting the underrepresentation of the population. Lastly, other antioxidants such as glutathione peroxidase and catalase were not measured in this study, which limits our ability to comprehensively evaluate the antioxidant defense capacity. Therefore, the findings should be interpreted with caution.

## 5. Conclusion

In summary, our findings suggest an independent association between decreased SOD activity and MS as well as possible mediating effect of impaired insulin sensitivity and *β*-cell dysfunction on the relationship between decreased SOD activity and MS. These observations provide proposed mechanistic insights into the underlying pathways explaining the relationship between decreased SOD activity and MS, which have not yet been fully understood. Nevertheless, further investigations are needed to validate our findings.

## Figures and Tables

**Table 1:** Differences of baseline characteristics between individuals with and without metabolic syndrome.

Variables	Non-MS (*n* = 247)	MS (*n* = 286)	*P*
Gender (male (%))	106 (42.9%)	85 (29.7%)	0.002
Age (years)	50.38 ± 11.88	54.33 ± 10.26	<0.001
BMI (kg/m^2^)	24.46 ± 3.22	27.48 ± 3.62	<0.001
WC (cm)	83.01 ± 8.81	90.73 ± 8.98	<0.001
HC (cm)	87.96 ± 9.56	95.72 ± 9.64	<0.001
WHR	0.94 (0.93, 0.96)	0.95 (0.93, 0.96)	0.244
SBP (mmHg)	121.22 ± 15.16	133.26 ± 17.63	<0.001
DBP (mmHg)	74.68 ± 9.06	77.55 ± 10.59	0.001
HbA1c (%)	5.40 (5.20, 5.70)	5.80 (5.50, 6.20)	<0.001
FPG (mmol/L)	5.59 (5.27, 6.07)	6.23 (5.80, 7.10)	<0.001
PG30 (mmol/L)	9.71 (8.30, 11.36)	11.29 (9.75, 13.69)	<0.001
PG60 (mmol/L)	8.80 (6.83, 10.69)	10.79 (8.57, 15.06)	<0.001
PG120 (mmol/L)	6.55 (5.43, 7.63)	8.13 (6.78, 11.65)	<0.001
HOMA-IR	2.07 (1.51, 2.80)	3.35 (2.43, 5.16)	<0.001
ISIM	20.56 (14.32, 27.24)	11.93 (8.13, 16.58)	<0.001
HOMA-*β*	74.02 (50.75, 105.68)	78.26 (55.88, 120.34)	0.104
ISSI-2	118.21 (86.91, 157.45)	84.42 (50.79, 114.69)	<0.001
UA (*μ*mol/L)	279.58 ± 77.15	301.97 ± 82.34	0.001
TC (mmol/L)	5.37 ± 0.93	5.60 ± 1.05	0.009
TG (mmol/L)	1.10 (0.80, 1.39)	1.85 (1.36, 2.52)	<0.001
HDL-C (mmol/L)	1.39 ± 0.26	1.20 ± 0.22	<0.001
LDL-C (mmol/L)	2.69 ± 0.67	2.97 ± 0.70	<0.001
Calories (kcal)	1507.60 (1193.59, 1901.27)	1507.60 (1142.49, 1873.42)	0.903
Calories (kcal/kg)	23.55 (17.57, 28.87)	21.08 (15.45, 26.29)	0.001
Fat	38.97 (19.55, 63.47)	38.97 (18.20, 57.79)	0.228
Protein	41.98 (32.37, 51.89)	41.98 (30.84, 50.46)	0.542
Carbohydrate	237.49 (190.51, 311.33)	237.49 (185.56, 318.02)	0.896
Vitamin A	117.90 (36.49, 243.36)	117.90 (48.81, 204.83)	0.615
Carotene	346.85 (101.81, 818.27)	346.85 (123.36, 792.20)	0.523
Vitamin C	35.85 (19.97, 58.50)	35.85 (18.68, 56.58)	0.732
Vitamin E	14.91 (8.55, 29.11)	14.91 (8.17, 32.75)	0.884
Zinc	7.22 (5.59, 9.25)	7.22 (5.09, 9.11)	0.634
Selenium	23.78 (16.51, 32.91)	23.78 (16.63, 31.60)	0.682
Manganese	4.56 (3.43, 5.90)	4.56 (3.37, 6.18)	0.876

Abbreviations: MS: metabolic syndrome; BMI: body mass index; WC: waist circumference; HC: hip circumference; WHR: waist to hip ratio; SBP: systolic blood pressure; DBP: diastolic blood pressure; HbA1c: glycosylated hemoglobin A1c; FPG: fasting plasma glucose; PG30, 60, and 120: postload plasma glucose at 30 min, 60 min, and 120 min during OGTT; FINS: fasting insulin; INS30, 60, and 120: postload insulin at 30 min, 60 min, and 120 min during OGTT; ISIM: Matsuda insulin sensitivity index; ISSI-2: insulin secretion-sensitivity index-2; UA: uric acid; TC: total cholesterol; TG: total triglyceride; HDL-C: high-density lipoprotein cholesterol; LDL-C: low-density lipoprotein cholesterol.

**Table 2:** Stepwise multivariate logistic regressions with SOD activity predicting MS.

Models	OR (per SD change for SOD activity)	95% CI	*P*
Model 1	0.719	0.572-0.903	0.005
Model 2	0.721	0.569-0.914	0.007
Model 3	0.718	0.565-0.912	0.007
Model 4	0.750	0.576-0.978	0.034

Model 1: adjusted for gender, age, and BMI. Model 2: adjusted for model 1+dietary factors (dietary intake of calories per kilogram body weight, fat, vitamin A, carotene, vitamin C, vitamin E, zinc, selenium, and manganese). Model 3: adjusted for model 2+cellular aging and oxidative stress markers (LTL, mtDNAcn, 8-OHdG, and GR). Model 4: adjusted for model 3+ISIM+ISSI-2. Abbreviations: OR: odds ratio; 95% CI: 95% confidence intervals; BMI: body mass index; LTL: leukocyte telomere length; mtDNAcn: mitochondrial DNA copy number; 8-OHdG: 8-hydroxy-2-deoxyguanosine; SOD: superoxide dismutase; GR: glutathione reductase; ISIM: Matsuda insulin sensitivity index; ISSI-2: insulin secretion-sensitivity index-2; MS: metabolic syndrome.

## Data Availability

The SPSS Statistics data used to support the findings of this study are available from the corresponding author upon request.
